# Drug repurposing against breast cancer by integrating drug-exposure expression profiles and drug–drug links based on graph neural network

**DOI:** 10.1093/bioinformatics/btab191

**Published:** 2021-03-19

**Authors:** Chen Cui, Xiaoyu Ding, Dingyan Wang, Lifan Chen, Fu Xiao, Tingyang Xu, Mingyue Zheng, Xiaomin Luo, Hualiang Jiang, Kaixian Chen

**Affiliations:** Drug Discovery and Design Center, State Key Laboratory of Drug Research, Shanghai Institute of Materia Medica, Chinese Academy of Sciences, Shanghai 201203, China; University of Chinese Academy of Sciences, Beijing 100049, China; Drug Discovery and Design Center, State Key Laboratory of Drug Research, Shanghai Institute of Materia Medica, Chinese Academy of Sciences, Shanghai 201203, China; University of Chinese Academy of Sciences, Beijing 100049, China; Drug Discovery and Design Center, State Key Laboratory of Drug Research, Shanghai Institute of Materia Medica, Chinese Academy of Sciences, Shanghai 201203, China; University of Chinese Academy of Sciences, Beijing 100049, China; Drug Discovery and Design Center, State Key Laboratory of Drug Research, Shanghai Institute of Materia Medica, Chinese Academy of Sciences, Shanghai 201203, China; University of Chinese Academy of Sciences, Beijing 100049, China; Drug Discovery and Design Center, State Key Laboratory of Drug Research, Shanghai Institute of Materia Medica, Chinese Academy of Sciences, Shanghai 201203, China; University of Chinese Academy of Sciences, Beijing 100049, China; Tencent AI Lab, Shenzhen 518057, China; Drug Discovery and Design Center, State Key Laboratory of Drug Research, Shanghai Institute of Materia Medica, Chinese Academy of Sciences, Shanghai 201203, China; University of Chinese Academy of Sciences, Beijing 100049, China; Drug Discovery and Design Center, State Key Laboratory of Drug Research, Shanghai Institute of Materia Medica, Chinese Academy of Sciences, Shanghai 201203, China; University of Chinese Academy of Sciences, Beijing 100049, China; Drug Discovery and Design Center, State Key Laboratory of Drug Research, Shanghai Institute of Materia Medica, Chinese Academy of Sciences, Shanghai 201203, China; University of Chinese Academy of Sciences, Beijing 100049, China; School of Life Science and Technology, ShanghaiTech University, Shanghai 200031, China; Drug Discovery and Design Center, State Key Laboratory of Drug Research, Shanghai Institute of Materia Medica, Chinese Academy of Sciences, Shanghai 201203, China; University of Chinese Academy of Sciences, Beijing 100049, China; School of Life Science and Technology, ShanghaiTech University, Shanghai 200031, China

## Abstract

**Motivation:**

Breast cancer is one of the leading causes of cancer deaths among women worldwide. It is necessary to develop new breast cancer drugs because of the shortcomings of existing therapies. The traditional discovery process is time-consuming and expensive. Repositioning of clinically approved drugs has emerged as a novel approach for breast cancer therapy. However, serendipitous or experiential repurposing cannot be used as a routine method.

**Results:**

In this study, we proposed a graph neural network model GraphRepur based on GraphSAGE for drug repurposing against breast cancer. GraphRepur integrated two major classes of computational methods, drug network-based and drug signature-based. The differentially expressed genes of disease, drug-exposure gene expression data and the drug–drug links information were collected. By extracting the drug signatures and topological structure information contained in the drug relationships, GraphRepur can predict new drugs for breast cancer, outperforming previous state-of-the-art approaches and some classic machine learning methods. The high-ranked drugs have indeed been reported as new uses for breast cancer treatment recently.

**Availabilityand implementation:**

The source code of our model and datasets are available at: https://github.com/cckamy/GraphRepur and https://figshare.com/articles/software/GraphRepur_Breast_Cancer_Drug_Repurposing/14220050.

**Supplementary information:**

Supplementary data are available at *Bioinformatics* online.

## 1 Introduction

Breast cancer is one of the most common cancer in women. According to statistics in 2018, more than 2 million new cases of breast cancer were identified, of which 0.6 million cases died. It accounted for about 15% of all cancer deaths among women worldwide (Bray *et al.*, 2018). The molecular profiling of breast cancer is heterogenous, which can be classified based on the expression of estrogen receptor (ER) or progesterone receptor, and human epidermal growth factor receptor 2 (Her2) (Hondermarck *et al.*, 2001). Thus, while tremendous resources are being invested in treatment, drugs approved for breast cancer therapy are costly and produce numerous side effects which are unbearable for the patients (Waks and Winer, 2019). It is necessary to develop more drugs to treat breast cancer.

The traditional discovery process for a new drug is time-consuming and expensive. It usually takes 10–15 years and 0.8–1.5 billion dollars, and has a high loss rate (Parvathaneni *et al.*, 2019). Many drug candidates have failed in early clinical trials due to side effects or poor efficacy (Arrowsmith, 2011). Drug repurposing, also known as drug repositioning, refers to a method that identifies new indications for approved drugs or drug candidates which have failed in the development phase (Parvathaneni *et al.*, 2019). Compared to traditional drug discovery process, drug repurposing may reduce the drug development period to 6.5 years and the research and development costs to $300 million (Pritchard *et al.*, 2017). Inspired by some successful cases, such as repurposing of thalidomide and sildenafil, funding for drug repurposing projects has increased substantially from 2012 to 2017 (Polamreddy and Gattu, 2019). Overall, the discovery of drug repurposing has been serendipitous or experiential historically. However, serendipity cannot be used as a routine method. With the rapid growth of computing power and data pertinent to drug repurposing, computational methods play an important role in drug repurposing studies by utilizing cheminformatics, bioinformatics and systems biology, computational methods.

Previous methods used for drug repurposing can be broadly separated into two categories: network-based and signature-based. The networks can be constructed based on drug–drug links information, which include the similarity, interaction or linkages between drugs, diseases and targets. Some studies defined the descriptors for each drug–disease pairs based on the similarities or relationships between drugs and diseases, and then constructed logistic regression model or statistical model to predict new drug–disease association (Gottlieb *et al.*, 2011; Iwata *et al.*, 2015). Cheng *et al.* presented a powerful network-based drug repurposing tool, Genome-wide Positioning Systems network (GPSnet). The GPSnet could predict drug responses in cancer cell lines accurately by integration with transcriptome profiles, whole-exome sequencing, drug–target network and drug-induced microarray data into human protein–protein interactome (Cheng *et al.*, 2019). Several studies inferred new drug indications by information flow or random walks on the networks which were built by these relationships mentioned above (Liu *et al.*, 2016; Luo *et al.*, 2016; Wang *et al.*, 2014). Xuan *et al.* integrated diverse prior knowledge of drugs and diseases through non-negative matrix factorization and then made prediction according to their projections of in low-dimensional feature space (Xuan *et al.*, 2019). Individualized Network-based Co-Mutation is a network-based approach for quantifying the putative genetic interactions in cancer. It can promote comprehensive identification of candidate therapeutic pathways (Liu *et al.*, 2020). Cheng *et al.* developed an integrative network-based infrastructure to identify potential targets or new indications for existing drugs by directly targeting significantly mutated genes or their neighbors in the protein interaction network (Cheng *et al.*, 2016). Drug repurposing can also be modeled as a problem of adjacency matrix completion as drugs and diseases networks can be represented by adjacency matrixes. Several methods have been proposed to build drug–disease networks based on known drug–disease relationships and then complement the adjacency matrixes of the networks with different algorithms (Luo *et al.*, 2018; Yang *et al.*, 2019a, b).

The signature-based methods have been successfully applied in the field of drug discovery, especially in precision medicine (Antman and Loscalzo, 2016; Dugger *et al.*, 2018). With advances in microarray and next-generation sequencing techniques, massive amounts of genomics data are accumulated. The Connectivity Map (CMap) contains many gene expression signatures from perturbation, which can be used to explore functional connections between diseases, genes and therapeutics (Lamb *et al.*, 2006). Dönertaş *et al.* identified repurposing for prolongevity drugs by comparing changes in gene expression with drug-perturbed expression profiles in the Connectivity Map (Donertas *et al.*, 2018). As the successor of CMap, the Library of Integrated Network-Based Cellular Signatures (LINCS) project consists of assay results from primary human cells treated with or without bioactive small molecules, ligands or genetic perturbations (Subramanian *et al.*, 2017). Drugs and their indications often share common related genes on which drugs execute their functions. The more common genes shared by a drug and disease, the more likely the drug is to be associated with the disease. Some studies have been proposed to infer the association of drugs and diseases based on their related genes or gene expressions (Saberian *et al.*, 2019; Sirota *et al.*, 2011). Analogously, some methods have been proposed according to the protein complexes shared by the drug and disease (Yu *et al.*, 2015) and their common perturbed genes (Peyvandipour *et al.*, 2018). However, the signature-based methods cannot be applied to the drugs and diseases without common related genes or proteins. In general, these two kinds of methods have their advantages and disadvantages. Network-based methods integrate the relationship between drugs but ignore prior knowledge. The signature-based methods leverage the characteristics of drugs or diseases themselves but cannot utilize the potential mechanisms included in drug–drug links information. These two methods have complementary advantages in drug repurposing studies.

In this study, we proposed GraphRepur, a prediction model for drug repurposing based on graph neural network. The model integrated two categories of computational methods mentioned above to take their advantages. We collected the drug-exposure gene expression data from LINCS project, and the drug–drug links information from STITCH database. To obtain the signature of drugs, we analyzed the differentially expressed genes for breast cancer. The drug-exposure gene expression from LINCS were used as drug signatures. Based on the drug–drug links information from STITCH database and drug signatures, a drug–drug links graph has been constructed, with drug signatures as the node features. GraphRepur took drug signatures and drug–drug links information as inputs and then output repurposing score of each drug for breast cancer. To benchmark the performance of GraphRepur, we compared the results to other deep learning and machine learning methods: deepDR (network-based) (Zeng *et al.*, 2019), LLE-DML (signature-based) (Saberian *et al.*, 2019), BiFusion (network-based) (Wang *et al.*, 2020), Graph Convolutional Networks (GCN) (Kipf and Welling, 2017), Graph Attention Networks (GATs) (Petar Veličković, 2017), Deep Neural Networks (DNNs) (LeCun *et al.*, 2015), Random Forest (Breiman, 2001), Support Vector Machines (SVMs) (Cortes and Vapnik, 1995) and Gradient Boosting Machines (GBMs) (Friedman, 2001) methods. Overall, GraphRepur can predict new drugs for breast cancer with area under the receiver operator characteristics curve (AUROC) and area under the precision recall curve (AUPR) significantly higher than the other methods on 5-fold cross validation. On external validation set, some of our predictions have been confirmed by studies published.

## 2 Materials and methods

### 2.1 Dataset

**Fig. 1.**
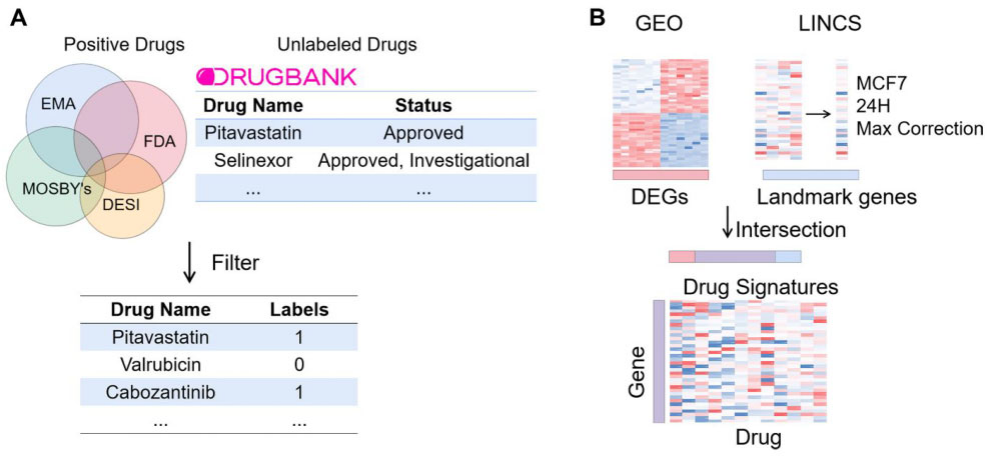
(**A**) The pipeline of dataset construction. (**B**) The pipeline of drug signatures generation

All drugs with development status of ‘Approved’ from DrugBank (V5.1.5) database were collected (Wishart *et al.*, 2018). Only drugs with drug-exposure gene express information in LINCS were retained. Among them, drugs which involved breast cancer were used as ‘positive drugs’ according to PharmaPendium (www.pharmapendium.com). PharmaPendium is a database of providing drug regulatory documents, adverse event, comparative safety, pharmacokinetic, efficacy, and metabolizing enzyme and transporter data. All the remaining drugs were used as ‘unlabeled drugs’. Finally, 25 in all 844 drugs are positive drugs in training set. For the external validation set, the drugs collected from PharmaPendium and DrugBank were taken intersection with LINCS project phase II, leading to 7 in 169 drugs are positive drugs in the external validation set. All chemical names, generic names, trade names or specific database ids of these drugs were converted to PubChem CID. The construction of dataset is shown in Figure 1A. All drugs are shown in Supplementary Table S1.

### 2.2 Differentially expressed genes

The gene expression microarray data of breast cancer was obtained from NCBI GEO database (ID: GSE26910). The measurements were performed on an Affymetrix Human Genome U133 Plus2.0 array plate. The preprocessing procedure we used included log2 transformation and quantile normalization (Irizarry *et al.*, 2003). The corresponding log2 (fold change) was calculated which is a ratio between the disease and control expression levels. For each gene, the *P*-value was calculated by a moderated *t*-test. Here, 2960 differentially expressed genes (*P* < 0.05) were obtained by comparing the gene expression levels between 6 stroma surrounding invasive breast primary tumors and 6 matched samples of normal stroma.

### 2.3 Drug signatures

The drug-exposure gene expression profiles of the differentially expressed genes in breast cancer cells were used as the drugs signatures. The drug-exposure gene expression data was obtained from LINCS project. LINCS phase I data was available in GEO Series GSE92742, and LINCS phase II data was available in GEO Series GSE70138. The LINCS data we used was level 5 data. The entries with MCF7 cell line, the time point of 24 h, the doses with highest replicate correlation coefficient were used as drug-exposure gene expression characteristics of drugs. The 978 landmark genes of LINCS data were screened for 2960 differentially expressed genes. Finally, drug signatures consisted of 199 drug-exposure gene expression profiles. The generation of drug signatures shown in Figure 1B.

### 2.4 Graph construction

Drug–drug links information has been widely used to study various drug-related problems, such as anatomical therapeutic chemical (ATC) classifiers of drugs (Chen *et al.*, 2014; Cheng *et al.*, 2017a,b; Chen and Jiang, 2015; Zhou *et al.*, 2019), adverse reactions prediction (Mayr *et al.*, 2018; Xian *et al.*, 2018; Zhao *et al.*, 2019) and targets prediction (Keiser *et al.*, 2007; Reker *et al.*, 2014; Wan *et al.*, 2019). Here, links information between drugs was identified according to the ‘combination score’ from STITCH (Szklarczyk *et al.*, 2015), which include drug–drug interaction, similarity and activity. The drug–drug links graph was constructed based on datasets, drug signatures and drug–drug links mentioned above. The nodes in the graph indicate drugs and the edges indicate interaction relationships between drugs. The node features in the graph are the drug signatures in Section 2.3. The interaction graph consists of 844 nodes and 20037 edges. The interaction relationships are shown in Supplementary Table S2.

### 2.5 Algorithm

GraphRepur was built based on GraphSAGE (Hamilton *et al.*, 2017), and modified the loss function of GraphSAGE to handle the imbalance data (detailed in Section 2.6). GraphSAGE included a set of aggregators. Each aggregator function learned to aggregate information from drug node signatures, topological structure of each drug node’s neighborhood and the distribution of drug node signatures in the neighborhoods. Compared to original GCN, GraphSAGE-based GraphRepur can be generalized to unseen nodes, since full graph laplacian was replaced with learnable aggregation functions. In the learning process, the model samples a given drug node’s local K-hop neighborhoods with fixed-size (K means the search depth). The embedding of given drug node was derived by aggregating node’s neighborhoods signatures, then was propagated to the next layer. During testing, the trained model generated embeddings of unseen drug nodes by applying the learned aggregation function. There are different aggregator functions which can be used in the aggregation step:

MEAN aggregator:

(1) $$h_{N(v)}^{k}\leftarrow \text{mean}\left(\left\{h_{u}^{k-1},u\in N(v)\right\}\right),$$

 

(2) $$h_{v}^{k}\leftarrow \sigma (W^{k}\cdot \text{CONCAT}(h_{v}^{k-1},h_{N(v)}^{k})).$$

GCN aggregator:

(3) $$h_{v}^{k}\leftarrow \sigma (W\cdot \text{mean}(\left\{h_{v}^{k-1}\right\}\cup \left\{h_{N(v)}^{k},\forall u\in N(v)\right\})).$$

LSTM aggregator: LSTMs was operated on a random permutation of node’s neighbors, since LSTMs are not inherently symmetric.

MaxPool aggregator:

(4) $$h_{N(v)}^{k}\leftarrow \text{max}\left(\left\{\sigma (W_{\text{pool}}h_{u_{i}}^{k-1}+b),\forall u_{i}\in N(v)\right\}\right),$$

 

(5) $$h_{v}^{k}\leftarrow \sigma (W^{k}\cdot \text{CONCAT}(h_{v}^{k-1},h_{N(v)}^{k})).$$

MeanPool aggregator:

(6) $$h_{N(v)}^{k}\leftarrow \text{mean}\left(\left\{\sigma (W_{\text{pool}}h_{u_{i}}^{k-1}+b),\forall u_{i}\in N(v)\right\}\right),$$

**Fig. 2.**
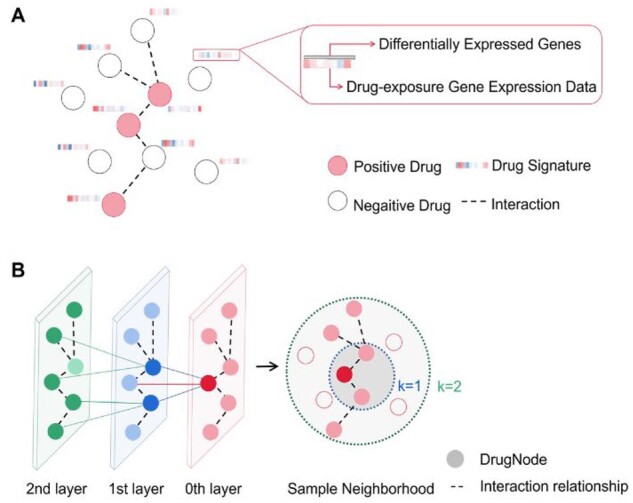
(**A**) Schematic of the data structure of GraphRepur. (**B**) Schematic of the GraphRepur sample and aggregate approach

 

(7) $$h_{v}^{k}\leftarrow \sigma (W^{k}\cdot \text{CONCAT}(h_{v}^{k-1},h_{N(v)}^{k})),$$

where *k* denotes current search depth, *h^k^* is a node’s representation at this search depth, *h^k^*^*-*^^*1*^ is node’s previous layer representation, *N* represents neighborhood function, $h_{\mathrm{(N(v))}}^{k}$ denotes the aggregated neighborhood vectors of node *v*, *W^k^* is a set of weight matrices, σ represents the activation function and *CONCAT* represents the concatenation operation. The data structure and algorithm schematic of the GraphRepur is shown in Figure 2.

### 2.6 Loss function

In our datasets, the numbers of positive drugs are tiny. This imbalance data causes inefficient training and degenerates models (Lin *et al.*, 2017). To address the issue, Focal Loss, a loss function for dealing with class imbalance effectively, was applied in our model (Lin *et al.*, 2017). The focal loss is a dynamically scaled cross entropy loss. The formula of focal loss is shown in Equation 8.

(8) $$L_{f1}=-\alpha y{\left(1-y^{\prime }\right)}^{\gamma }\;\text{log}\;y^{\prime }-(1-\alpha )(1-y){y^{\prime }}^{\gamma }\;\text{log}(1-y^{\prime }),$$

where y and $y^{\prime }$ are true label and predicted results, just like cross entropy, and γ is a tunable focusing parameter, ${\left(1-y^{\prime }\right)}^{\gamma }$ is a modulating factor to the cross entropy, α is a weighting factor, range from 0 to 1. When the focusing parameter γ was 0 and the weighting factor α was 0.5, the focal loss was the same as the cross-entropy.

### 2.7 Method comparison

In order to compare the performance of GraphSAGE with different aggregators and loss functions by grid search (detailed in Section 3.1 and Supplementary Table S3), we selected the best model for prediction drug repurposing against breast cancer, named as GraphRepur. To evaluate the performance of the model, we compared the GraphRepur with graph conventional network, DeepDR, BiFusion, LLE-DML, GATs, DNN s and three machine learning methods. All models have been optimized by grid searching to adjust hyperparameters. The range of considered hyperparameters for GraphSAGE is shown in Table 1.

**Table 1.** Hyperparameters space considered

Hyperparameter	Values considered
Loss function	Focal (*α* = 0.75, *γ* = 2); Focal (*α* = 0.25, *γ* = 2); Cross-Entropy
Hidden units	(32,32); (64,32); (64,64); (128,64); (128,128); (256,128); (256,256); (512,256)
Sampling number	(5,4,0); (8,5,0); (12,8,0); (16,9,0); (20,10,0); (25,10,0)
Learning rate	0.01; 0.005; 0.001; 0.0005; 0.0001
Dropout	0.2; 0.4
Batch size	64; 128; 256; 512

GCN. GCN is a semi-supervised classification on graph-structured data which generalizes the operation of convolution from traditional data (images or grids) to graph data. The considered hyperparameters are shown in Supplementary Table S4.

DeepDR is a network-based approach for drug repurposing prediction. This approach learned high-level features of drugs from ten networks including one drug–disease, one drug-side-effect, one drug–target and seven drug–drug networks by a multi-modal deep autoencoder. To infer candidates for approved drugs, the representation of drugs together with clinically reported drug–disease pairs are encoded and decoded through a variational autoencoder. DeepDR outperformed conventional network-based or machine learning-based approaches on a balance dataset in original literature. For benchmarking, the deepDR with and without fine tuning were applied on our dataset. The adjustable hyperparameters are shown in Supplementary Table S5.

BiFusion is a bipartite graph convolution network model through heterogeneous information fusion which includes interactions between diverse biological domains. BiFusion outperformed conventional network-based such as GCN, DeepWalk and cVAE on repoDB dataset. Here, BiFusion were applied on our dataset. The adjustable hyperparameters are shown in Supplementary Table S6.

LLE-DML is a signature-based approach which incorporated a non-linear dimensionality reduction algorithm (LLE) with the distance metric learning (DML) algorithm (Saberian *et al.*, 2019). Based on LLE-DML, disease gene expression profiles, large-scale drug-exposure gene expression profiles and the clinical established knowledge have been transformed into a space in which the disease–drug pairs clinically effective become closer to each other.

GAT. GAT is a spatial-based graph convolution network which incorporates the attention mechanism into the propagation step. The attention mechanism is involved in determining the weights of a node’s neighbors when aggregating feature information. The considered hyperparameters are shown in Supplementary Table S7.

DNN. DNN is a classical deep learning model. The considered hyperparameters are shown in Supplementary Table S8.

SVM. Two hyperparameters were considered: the penalty parameter C kernel type and kernel coefficient, with their ranges is shown in Supplementary Table S9.

Random Forest. The considered hyperparameters are shown in Supplementary Table S10.

GBMs. The considered hyperparameters are shown in Supplementary Table S11.

GCN, GraphSAGE, GAT and DNN models were developed in Tensorflow (version 1.14.0) and were performed on standard NVIDIA GPUs. DeepDR was developed in torch (version 1.4.0). BiFusion was developed in torch (version 1.5.0). LLE-DML was developed in R (version 3.5.3). All machine learning models were implemented by using scikit-learn (Pedregosa *et al.*, 2011). All code was implemented using Python 3.5.4.

## 3 Results

### 3.1 Model optimization and performance

To optimize the model, the dataset was divided into five sets, we selected one of which as validation set, one as test set and the remaining three as training set. The rectified linear unit was used as the activation function for each layer except the output layer. We explored different hyperparameters through a grid search, including hidden units, sampling number, learning rate, loss function parameters, L2 regularization, dropout rate and batch size. In order to prevent overfitting, the loss on the validation set was monitored by early-stopping. The training was stopped once the loss did not decrease 30 times continuously. The range of considered hyperparameters is shown in Table 1. We considered two hidden layers with 32, 64, 128 or 256 neurons in each hidden layer. The Adam optimizer with initial learning rates of 0.01, 0.005, 0.001, 0.0005 and 0.0001 was used as optimizer. We used dropout and early-stopping for regularization during the process of training. The best models with different loss function and aggregators were selected according to the performance on validation set. The performance on test set based on AUROC and AUPR is shows in Supplementary Table S3. All performance on the testing dataset of the GraphRepur-GCN models is shown in Supplementary Table S12.

We compared the GraphRepur based on different aggregators with graph conventional network, DeepDR, BiFusion, LLE-DML, GATs, deep neural networks, random forest, SVMs and GBMs. Among them, the input of deep neural network, BiFusion, random forest, SVM and GBM was just the drug signatures. In order to compare the performance of different methods, we provided performance measures that were typical for classification: AUROC and AUPR. The average performance of 5-fold cross-validation for all models is shown in Table 2.

**Table 2.** Methods comparison based on AUROC and AUPR

Method	AUROC	AUPR
GraphRepur	**0.81 ± 0.03**	**0.59 ± 0.06**
Graph Convolution Network	0.75 ± 0.20	0.38 ± 0.15
DeepDR	0.53 ± 0.11	0.07 ± 0.02
DeepDR (fine tuned)	0.56 ± 0.12	0.08 ± 0.03
BiFusion	0.63 ± 0.07	0.21 ± 0.18
Graph Attention Network	0.74 ± 0.11	0.54 ± 0.02
LLE-DML	0.69 ± 0.01	0.28 ± 0.001
Deep Neural Network	0.64 ± 0.04	0.16 ± 0.08
Support Vector Machine	0.62 ± 0.11	0.12 ± 0.08
Random Forest	0.66 ± 0.15	0.09 ± 0.09
Gradient Boosting Machine	0.63 ± 0.12	0.11 ± 0.08

*Note:* All values are mean values ± one standard deviation. The best performance is shown in bold.

Among these models, the GraphRepur has the best-performing with an AUROC and AUPR of 0.81 and 0.59, respectively. The graph conventional network and GATs models were slightly worse. Overall, the performance using the graph neural networks were better than the DNN and machine learning, that models using drug signatures only, especially in AUPR.

### 3.2 Analysis of the contribution of drug–drug links

Gene expression profile is a kind of classic Euclidean data which can be used as input of machine learning methods and deep neural networks. Graph, also known as network, is a kind of non-Euclidean structure data which consists of a set of nodes and edges. According to Section 3.1, the performance of models using non-Euclidean structure data, GraphRepur with different aggregators and GAT, was much better than the models using Euclidean structure data. It might suggest that drug–drug links data provide extremely important information in drug repurposing prediction.

To verify this guess, we compared the proportion of positive drugs (PPD) in the first-order and second-order neighbor node of positive and unlabeled drugs. As shown in Table 3, the PPD of positive drugs was 0.1747 in the first-order neighbor, and the PPD of unlabeled drugs was 0.0291. The *P*-value was $4.302\times 10^{-7}$. The PPD of positive drugs was 0.0408 in second-order neighbor, and the PPD of unlabeled drug was 0.0329. The *P* value was 0.0016. In both the first-order and the second-order neighbor, the PPDs of positive drugs were significantly higher than the PPD of unlabeled drugs. It suggested that drug neighbor nodes in drug–drug links networks provided important clues for drug repurposing prediction.

**Table 3.** The proportion of positive drugs (PPD) in drug neighbors

Item	Positive	Unlabeled	*P*-value
First-order neighour PPD	0.1747 ± 0.10	0.0291 ± 0.06	4.302E-07
Second-order neighour PPD	0.0408 ± 0.01	0.0329 ± 0.01	0.0016

To investigate the problem further, GraphRepur was applied on a random links network which the average node degree was the same as the true links network. The hyperparameters range was the same as in Section 3.1. The model was evaluated using 5-fold cross validation, and the best performing AUROC and AUPR was 0.654 and 0.513, respectively. The performance of random links model was worse than the real links model. It suggested that the drug–drug links information was helpful in researching drug repurposing.

### 3.3 Analysis of the contribution of different links types

STITCH provides five types of drug–drug links relationships, namely ‘Similarity’, ‘Experiment’, ‘Database’, ‘TextMining’ and ‘Combined Score’. The first four items were assessed by association of the structure, activity, reactions and co-occurrence in literatures. The last item ‘Combined Score’, the links type we used, was an integrated evaluation based on the first four items. To explore the contribution of each links type for drug repurposing, we compared the performance of these five type links by using the GraphRepur model. In addition, GraphRepur was applied on five random links networks which the average node degrees were the same as the true links networks. The hyperparameters range was the same as in Section 3.1. Each type was evaluated using 5-fold cross validation. The results are shown in Supplementary Table S13. It can be found that all the performance of random networks was worse than the true network. It suggested that real links relationships provided important information in prediction. Furthermore, in the performance of real links, we found that three worse performance types (‘similarity’, ‘experimental’ and ‘database’) had much lower average node degrees than the better performance types (‘text mining’ and ‘combined score’). Moreover, the number of isolated nodes in the worse performance types was much more than in better performance types. It suggested that the worse performance types had sparser information distribution, and thus the topology information they provided was limited. The characteristics of different type links graphs are shown in Supplementary Table S14, and violin plots of degree distribution for links types are shown in Supplementary Figure S1.

### 3.4 Analysis of the contribution of drug signatures

Although the drug–drug links information provides important clues, it is not sufficient to ignore the gene expression profiling. To evaluate the importance of drug signatures, we used the GraphRepur model to make predictions on a dataset which contained just links information without drug signatures. The hyperparameters range was the same as in Section 3.1. These links networks were evaluated using 5-fold cross validation, and the best performing AUROC and AUPR was 0.587 and 0.505, respectively. The performance of the model containing just links information is worse than that of the model containing both links information and gene expression information.

### 3.5 External validation set evaluation

Despite GraphRepur performs well on the internal test dataset, it is necessary to evaluate the generalization ability of model on external test set. Here, we built an external validation set from LINCS II. The external validation set include seven positive drugs for breast cancer. GraphRepur were used in prediction of the external validation set. The hyperparameters of all models were obtained from the best performance in Section 3.1. Five sub-models trained on the 5-fold cross validation were used in the external validation set. The performance of each sub-model on the external test set is shown in Supplementary Table S15. The prediction results of the GraphRepur model on the external validation set are shown in Supplementary Table S16.

In all 169 prediction results, drugs which not approved for breast cancer in the top 30 were searched in clinicaltrial.gov and PubMed. The GraphRepur predictions supporting by literature evidence are shown in Table 4. Eight of these drugs are undergoing in clinical trials for breast cancer, involving more than 40 clinical trials. The clinical trials for breast cancer in all 169 predictions are shown in Supplementary Table S17. Of all the 169 external validation set drugs, only 30 drugs had clinical trials with ‘Completed’ or ‘Active’ status, of which 12 were in the top 30.

**Table 4.** New drugs predictions for breast cancer

Rank	Drug name	Origin indication	Supported literature
4	Selinexor	Refractory Multiple Myeloma	Shafique *et al.* (2019)
5	Mycophenolic acid	Kidney Transplant Rejection	Aghazadeh and Yazdanparast (2016).
8	Pitavastatin	Primary Hyperlipidemia	Kubatka *et al.* (2014)
12	Etravirine	Human Immunodeficiency Virus type 1 infection	Reznicek *et al.* (2016)
13	Idelalisib	Chronic Lymphocytic Leukemia; Relapsed Follicular B-cell non-Hodgkin Lymphoma; Relapsed Small Lymphocytic Lymphoma	Alipour *et al.* (2019)
14	Dimethyl fumarate	Multiple Sclerosis	Kastrati *et al.* (2016)
17	Bazedoxifene	Severe Vasomotor Symptoms	Fabian *et al.* (2019)
21	Ibrutinib	Chronic Lymphocytic Leukemia; Mantle Cell Lymphoma; Waldenström's Macroglobulinemia	Varikuti *et al.* (2020)
23	Vismodegib	Locally Advanced or Metastatic Basal Cell Carcinoma	Valenti *et al.* (2017)
24	Sunitinib	Advanced Renal Cell Carcinoma	Korashy *et al.* (2017).

Selinexor is a selective inhibitor of nuclear transport (SINE). It is approved for the treatment of multiple myeloma. In clinical studies (ClinicalTrials.gov Identifier: NCT02402764, NCT02025985), selinexor was fairly well tolerated in patients with advanced triple negative breast cancer (TNBC), and the clinical benefit rate [CBR, Complete Response + Partial Response + stable disease (SD) ≥12 weeks] was 30% (Shafique *et al.*, 2019). In future studies, researchers will focus on the combination use of selinexor and identify the patients most likely to benefit with appropriate biomarker drivers (Shafique *et al.*, 2019). Mycophenolic acid (MPA) is an inhibitor of de novo guanine nucleotide synthesis with potential anti-cancer activity. A study suggested that MPA might provide an alternative clinical strategy for chemosensitization of resistant breast cancer cells to anti-HER2 therapy (Aghazadeh and Yazdanparast, 2016). Pitavastatin, a lipid-lowering drug for primary hyperlipidemia or mixed dyslipidemia. Kubatka *et al.* found a partial antineoplastic effect of pitavastatin combined with melatonin in the rat mammary gland carcinoma model (Kubatka *et al.*, 2014). Wang *et al.* found that pitavastatin could suppress tumor growth in mouse model. Idelalisib (also known as CAL-101) is a phosphoinosine 3-kinase inhibitor for the treatment of chronic lymphocytic leukemia (CLL), recurrent follicular B-cell non-Hodgkin lymphoma (FL) and recurrent small lymphocytic lymphoma. Alipour *et al.* found that idelalisib could considerably decrease the viability of both ER-positive MCF-7 and triple negative MDA-MB-468 cells (Alipour *et al.*, 2019). Dimethyl fumarate (DMF) is an anti-inflammatory drug for multiple sclerosis patients. Kastrati *et al.* found that DMF had anti-cancer stem cell properties by effectively blocking NFκB activity in multiple breast cancer cell lines and abrogating NFκB-dependent mammosphere formation (Kastrati *et al.*, 2016). Ibrutinib is Bruton tyrosine kinase inhibitor for treating CLL and Mantle cell lymphoma. Some studies found that ibrutinib could inhibit tumor development and metastasis in breast cancer (Varikuti *et al.*, 2020). Vismodegib is a hedgehog signaling pathway inhibitor for treatment of adult basal cell carcinoma. Valenti *et al.* found that vismodegib may offer a novel therapeutic strategy against breast cancer by reducing cancer-associated fibroblasts and subsets of cancer stem cells expansion (Valenti *et al.*, 2017). Sunitinib is a small molecule multi-target receptor tyrosine kinase inhibitor. Korashy *et al.* found that sunitinib could cause concentration-dependent cell growth suppression on MCF7 cells (Korashy *et al.*, 2017). These literatures above supported that the GraphRepur prediction could identify potentially effective drugs for breast cancer drugs, and GraphRepur had the potential to promote the research of drug repurposing.

### 3.6 Evaluation on different cell lines

Breast cancer is very heterogenous. In order to examine the performance of the model in different cell lines, we established two external validation sets based on BT-20 (ER-) and SK-BR3 (HER2-enriched) from LINCS. These external validation sets include 3 positive drugs for breast cancer. The hyperparameters of GraphRepur were obtained from the best performance in Section 3.1. Five sub-models trained on the 5-fold cross validation were used in the external validation set. The average performance of each sub-model on these external test sets is shown in Supplementary Table S18. Compared to MCF7, the performance of GraphRepur on BT-20 and SK-BR-3 was a little worse, but it still had predictive ability. It suggested that GraphRepur had some capacity for prediction on different cancer subtype cell lines.

## 4 Discussion

Drug repurposing can identify new indications for approved drugs or drug candidates. It has various advantages such as cost effectiveness and shortened timeline. In this study, we established a graph neural network model, GraphRepur, to predict new drugs for breast cancer. GraphRepur integrated two major classes of computational methods for drug repurposing that the drug network-based and drug signature-based. We constructed a graph containing drug–drug links relationships and drug gene expression signatures. By extracting the drug signatures and topological structure information contained in the graph, we established a drug repurposing prediction model for breast cancer. By comparison, the GraphRepur achieved better performance at 5-fold cross validation. After that, we analyzed the reasons for the better performance of the graph neural network, discussed the contribution of drug gene expression information, compared the performance of different links types and discussed the reasons. Finally, we evaluated the performance of the GraphRepur on external validation dataset. Some of our predictions are confirmed by retrospective analyzing recently reported drug repurposing studies against breast cancer.

Non-Euclidean structural data graphs can integrate both drug relationships and genomics data. However, due to the irregular structure of non-Euclidean data, classical machine learning and deep learning algorithms are not applicable. The graph neural network has powerful graph representation capabilities, thus GraphRepur can combine the advantage of the two kinds of computational methods for drug repurposing.

There are limitations to the application scope of this study. GraphRepur cannot make predictions for diseases which lack known effective drugs, and thus cannot be used for orphan drugs discovery. While, the GraphRepur can be transformed to other diseases by retraining or fine tuning with relevant data. In addition, the unlabeled dataset used in this study was not associated with breast cancer according to PharmaPendium. But the real negative drugs are still lacking. The model will substantially benefit from more adequate and unambiguous negative data available in the future. Another limitation is tumor heterogeneity, which is a major barrier to understanding tumorigenesis, disease progression and the efficacy of therapy. Although we did have some discussion about it, the dataset about cancer subtypes was sorely lacking, whether cells or drugs, which hinders the further development of relevant researches. The creation and updating of databases containing cancer subtypes information will help researchers build more clinically useful models for various cancer subtypes in the future.

Panomics includes multidisciplinary research fields, such as genomics, epigenomics, proteomics, metabolomics and transcriptomics, etc. The drug exposure gene expression information we used belongs to a kind of ‘panomics’ data. Different panomics data provide different perspectives for researching biological processes. For example, single-cell sequencing and computational methods have made it possible to treat tumors by selectively targeting specific clones that mediate tumorigenesis or drug resistance (Cheng *et al.*, 2017a,b). Cheng *et al.* discussed the prospects for the application of panomics data used in drug repurposing in a review (Cheng *et al.*, 2017a,b). In the future, the combination of panomics data and artificial intelligence algorithms can further promote the research of drug repurposing.

## Author Contributions

X.L. and M.Z. designed the study and were responsible for the integrity of the manuscript. C.C., X.D. and D.W. performed the analysis and all calculations. L.C. contributed to the final code testing and correction. C.C. mainly wrote the manuscript. F.X. contributed to data processing. T.X. contributed to response. H.J. and K.C. gave conceptual advice. All authors discussed and commented on the manuscript.

## Supplementary Material

btab191_Supplementary_Data

## Data Availability

Publicly available datasets were analyzed in this study. This data can be found here: https://github.com/cckamy/GraphRepur.

## References

[btab191-B1] Aghazadeh S. , Yazdanparast R. (2016) Mycophenolic acid potentiates HER2-overexpressing SKBR3 breast cancer cell line to induce apoptosis: involvement of AKT/FOXO1 and JAK2/STAT3 pathways. Apoptosis Int. J. Programmed Cell Death, 21, 1302–1314. 10.1007/s10495-016-1288-4 27651367

[btab191-B2] Alipour F. et al (2019) Inhibition of PI3K pathway using BKM120 intensified the chemo-sensitivity of breast cancer cells to arsenic trioxide (ATO). Int. J. Biochem. Cell Biol., 116, 105615. 31539632 10.1016/j.biocel.2019.105615

[btab191-B3] Antman E.M. , Loscalzo J. (2016) Precision medicine in cardiology. Nat. Rev. Cardiol., 13, 591–602. 27356875 10.1038/nrcardio.2016.101

[btab191-B4] Arrowsmith J. (2011) Trial watch: phase III and submission failures: 2007-2010. Nat. Rev. Drug Discov., 10, 87. 21283095 10.1038/nrd3375

[btab191-B5] Bray F. et al (2018) Global cancer statistics 2018: GLOBOCAN estimates of incidence and mortality worldwide for 36 cancers in 185 countries. Cancer J. Clin., 68, 394–424. 10.3322/caac.21492 30207593

[btab191-B6] Breiman L. (2001) Random forests. Mach. Learn., 45, 5–32.

[btab191-B7] Chen L. et al (2014) A hybrid method for prediction and repositioning of drug anatomical therapeutic chemical classes. Mol. Biosyst., 10, 868–877. 24492783 10.1039/c3mb70490d

[btab191-B8] Chen F.S. , Jiang Z.R. (2015) Prediction of drug's Anatomical Therapeutic Chemical (ATC) code by integrating drug-domain network. J. Biomed. Inform., 58, 80–88. 26434987 10.1016/j.jbi.2015.09.016

[btab191-B9] Cheng F. et al (2016) A network-based drug repositioning infrastructure for precision cancer medicine through targeting significantly mutated genes in the human cancer genomes. J. Am. Med. Inf. Assoc. JAMIA, 23, 681–691. 10.1093/jamia/ocw007 PMC6370253 27026610

[btab191-B10] Cheng F. et al (2017a) Individualized network-based drug repositioning infrastructure for precision oncology in the panomics era. Brief. Bioinf., 18, 682–697. 10.1093/bib/bbw051 27296652

[btab191-B11] Cheng X. et al (2017b) iATC-mISF: a multi-label classifier for predicting the classes of anatomical therapeutic chemicals. Bioinformatics, 33, 341–346. 28172617 10.1093/bioinformatics/btw644

[btab191-B12] Cheng F. et al (2019) A genome-wide positioning systems network algorithm for in silico drug repurposing. Nat. Commun., 10, 3476. 31375661 10.1038/s41467-019-10744-6 PMC6677722

[btab191-B13] Cortes C. , Vapnik V. (1995) Support-vector networks. Mach. Learn., 20, 273–297.

[btab191-B14] Donertas H.M. et al (2018) Gene expression-based drug repurposing to target aging. Aging Cell, 17, e12819. 29959820 10.1111/acel.12819 PMC6156541

[btab191-B15] Dugger S.A. et al (2018) Drug development in the era of precision medicine. Nat. Rev. Drug Discov., 17, 183–196. 29217837 10.1038/nrd.2017.226 PMC6287751

[btab191-B16] Fabian C.J. et al (2019) Effect of bazedoxifene and conjugated estrogen (Duavee) on breast cancer risk biomarkers in high-risk women: a pilot study. Cancer Prev. Res. (PA), 12, 711–720. 10.1158/1940-6207.CAPR-19-0315 PMC6774863 31420361

[btab191-B17] Friedman J.H. (2001) Greedy function approximation: a gradient boosting machine. Ann. Stat., 29, 1189–1232.

[btab191-B18] Gottlieb A. et al (2011) PREDICT: a method for inferring novel drug indications with application to personalized medicine. Mol. Syst. Biol., 7, 496. 21654673 10.1038/msb.2011.26 PMC3159979

[btab191-B19] Hamilton W.L. et al (2017) Inductive representation learning on large graphs. In Proceedings of the 31st International Conference on Neural Information Processing Systems, pp. 1025–1035.

[btab191-B20] Hondermarck H. et al (2001) Proteomics of breast cancer for marker discovery and signal pathway profiling. Proteomics, 1, 1216–1232. 11721634 10.1002/1615-9861(200110)1:10<1216::AID-PROT1216>3.0.CO;2-P

[btab191-B21] Irizarry R.A. et al (2003) Exploration, normalization, and summaries of high density oligonucleotide array probe level data. Biostatistics, 4, 249–264. 12925520 10.1093/biostatistics/4.2.249

[btab191-B22] Iwata H. et al (2015) Systematic drug repositioning for a wide range of diseases with integrative analyses of phenotypic and molecular data. J. Chem. Inf. Model., 55, 446–459. 25602292 10.1021/ci500670q

[btab191-B23] Kastrati I. et al (2016) Dimethyl fumarate inhibits the nuclear factor B pathway in breast cancer cells by covalent modification of p65 protein. J. Biol. Chem., 291, 3639–3647. 26683377 10.1074/jbc.M115.679704 PMC4751401

[btab191-B24] Keiser M.J. et al (2007) Relating protein pharmacology by ligand chemistry. Nat. Biotechnol., 25, 197–206. 17287757 10.1038/nbt1284

[btab191-B25] Kipf T.N. , Welling M. (2017) Semi-supervised classification with graph convolutional networks. arXiv preprint arXiv:1609.02907.

[btab191-B26] Korashy H.M. et al (2017) Sunitinib inhibits breast cancer cell proliferation by inducing apoptosis, cell-cycle arrest and DNA repair while inhibiting NF-kappaB signaling pathways. Anticancer Res., 37, 4899–4909. 28870911 10.21873/anticanres.11899

[btab191-B27] Kubatka P. et al (2014) Combination of Pitavastatin and melatonin shows partial antineoplastic effects in a rat breast carcinoma model. Acta Histochem., 116, 1454–1461. 25450902 10.1016/j.acthis.2014.09.010

[btab191-B28] Lamb J. et al (2006) The Connectivity Map: using gene-expression signatures to connect small molecules, genes, and disease. Science, 313, 1929–1935. 17008526 10.1126/science.1132939

[btab191-B29] LeCun Y. et al (2015) Deep learning. Nature, 521, 436–444. 26017442 10.1038/nature14539

[btab191-B30] Lin T.-Y. et al (2017) Focal loss for dense object detection. In *IEEE International Conference on Computer Vision*, 1, pp. 2999–3007.

[btab191-B31] Liu C. et al (2020) Individualized genetic network analysis reveals new therapeutic vulnerabilities in 6,700 cancer genomes. PLoS Comput. Biol., 16, e1007701. 32101536 10.1371/journal.pcbi.1007701 PMC7062285

[btab191-B32] Liu H. et al (2016) Inferring new indications for approved drugs via random walk on drug–disease heterogenous networks. BMC Bioinformatics, 17, 539. 28155639 10.1186/s12859-016-1336-7 PMC5259862

[btab191-B33] Luo H.M. et al (2016) Drug repositioning based on comprehensive similarity measures and Bi-Random walk algorithm. Bioinformatics, 32, 2664–2671. 27153662 10.1093/bioinformatics/btw228

[btab191-B34] Luo H.M. et al (2018) Computational drug repositioning using low-rank matrix approximation and randomized algorithms. Bioinformatics, 34, 1904–1912. 29365057 10.1093/bioinformatics/bty013

[btab191-B35] Mayr A. et al (2018) Large-scale comparison of machine learning methods for drug target prediction on ChEMBL. Chem. Sci., 9, 5441–5451. 30155234 10.1039/c8sc00148k PMC6011237

[btab191-B36] Parvathaneni V. et al (2019) Drug repurposing: a promising tool to accelerate the drug discovery process. Drug Discov. Today, 24, 2076–2085. 31238113 10.1016/j.drudis.2019.06.014 PMC11920972

[btab191-B37] Pedregosa F. et al (2011) Scikit-learn: machine learning in Python. J. Mach. Learn. Res., 12, 2825–2830.

[btab191-B38] Petar Veličković G.C. et al (2017) Graph attention networks. arXiv, Preprint, arXiv:1710.10903.

[btab191-B39] Peyvandipour A. et al (2018) A novel computational approach for drug repurposing using systems biology. Bioinformatics, 34, 2817–2825. 29534151 10.1093/bioinformatics/bty133 PMC6084573

[btab191-B40] Polamreddy P. , Gattu N. (2019) The drug repurposing landscape from 2012 to 2017: evolution, challenges, and possible solutions. Drug Discov. Today, 24, 789–795. 30513339 10.1016/j.drudis.2018.11.022

[btab191-B41] Pritchard J.L.E. et al (2017) Enhancing the promise of drug repositioning through genetics. Front. Pharmacol., 8, 896. 29270124 10.3389/fphar.2017.00896 PMC5724196

[btab191-B42] Reker D. et al (2014) Identifying the macromolecular targets of de novo-designed chemical entities through self-organizing map consensus. Proc. Natl. Acad. Sci. USA, 111, 4067–4072. 24591595 10.1073/pnas.1320001111 PMC3964060

[btab191-B43] Reznicek J. et al (2016) Etravirine inhibits ABCG2 drug transporter and affects transplacental passage of tenofovir disoproxil fumarate. Placenta, 47, 124–129. 27780535 10.1016/j.placenta.2016.09.019

[btab191-B44] Saberian N. et al (2019) A new computational drug repurposing method using established disease-drug pair knowledge. Bioinformatics, 35, 3672–3678. 30840053 10.1093/bioinformatics/btz156 PMC6761937

[btab191-B45] Shafique M. et al (2019) A phase II trial of selinexor (KPT-330) for metastatic triple-negative breast cancer. Oncologist, 24, 887–e416. 30996012 10.1634/theoncologist.2019-0231 PMC6656474

[btab191-B46] Sirota M. et al (2011) Discovery and preclinical validation of drug indications using compendia of public gene expression data. Sci. Transl. Med., 3, 96ra77. 10.1126/scitranslmed.3001318 PMC3502016 21849665

[btab191-B47] Subramanian A. et al (2017) A next generation connectivity map: L 1000 platform and the first 1,000,000 profiles. Cell, 171, 1437–1452.e17. 29195078 10.1016/j.cell.2017.10.049 PMC5990023

[btab191-B48] Szklarczyk D. et al (2015) STRING v10: protein–protein interaction networks, integrated over the tree of life. Nucleic Acids Res., 43, D447–452. 25352553 10.1093/nar/gku1003 PMC4383874

[btab191-B49] Valenti G. et al (2017) Cancer stem cells regulate cancer-associated fibroblasts via activation of hedgehog signaling in mammary gland tumors. Cancer Res., 77, 2134–2147. 28202523 10.1158/0008-5472.CAN-15-3490

[btab191-B50] Varikuti S. et al (2020) Ibrutinib treatment inhibits breast cancer progression and metastasis by inducing conversion of myeloid-derived suppressor cells to dendritic cells. Br. J. Cancer, 122, 1005–1013. 32025027 10.1038/s41416-020-0743-8 PMC7109110

[btab191-B51] Waks A.G. , Winer E.P. (2019) Breast cancer treatment: a review. JAMA, 321, 288–300. 30667505 10.1001/jama.2018.19323

[btab191-B52] Wan F. et al (2019) NeoDTI: neural integration of neighbor information from a heterogeneous network for discovering new drug–target interactions. Bioinformatics, 35, 104–111. 30561548 10.1093/bioinformatics/bty543

[btab191-B53] Wang W.H. et al (2014) Drug repositioning by integrating target information through a heterogeneous network model. Bioinformatics, 30, 2923–2930. 24974205 10.1093/bioinformatics/btu403 PMC4184255

[btab191-B54] Wang Z. et al (2020) Toward heterogeneous information fusion: bipartite graph convolutional networks for in silico drug repurposing. Bioinformatics, 36, i525–i533. 32657387 10.1093/bioinformatics/btaa437 PMC7355266

[btab191-B55] Wishart D.S. et al (2018) DrugBank 5.0: a major update to the DrugBank database for 2018. Nucleic Acids Res., 46, D1074–D1082. 29126136 10.1093/nar/gkx1037 PMC5753335

[btab191-B56] Xian Z. et al (2018) A similarity-based method for prediction of drug side effects with heterogeneous information. Math. Biosci., 306, 136–144. 30296417 10.1016/j.mbs.2018.09.010

[btab191-B57] Xuan P. et al (2019) Drug repositioning through integration of prior knowledge and projections of drugs and diseases. Bioinformatics, 35, 4108–4119. 30865257 10.1093/bioinformatics/btz182

[btab191-B58] Yang M.Y. et al (2019a) Drug repositioning based on bounded nuclear norm regularization. Bioinformatics, 35, I455–I463. 31510658 10.1093/bioinformatics/btz331 PMC6612853

[btab191-B59] Yang M.Y. et al (2019b) Overlap matrix completion for predicting drug-associated indications. PLoS Comput. Biol., 15, e1007541. 31869322 10.1371/journal.pcbi.1007541 PMC6946175

[btab191-B60] Yu L. et al (2015) Inferring drug–disease associations based on known protein complexes. BMC Med. Genomics, 8, S2. 10.1186/1755-8794-8-S2-S2 PMC4460611 26044949

[btab191-B61] Zeng X. et al (2019) deepDR: a network-based deep learning approach to in silico drug repositioning. Bioinformatics, 35, 5191–5198. 31116390 10.1093/bioinformatics/btz418 PMC6954645

[btab191-B62] Zhao X. et al (2019) Predicting drug side effects with compact integration of heterogeneous networks. Curr. Bioinform., 14, 709–720.

[btab191-B63] Zhou J.P. et al (2019) iATC-NRAKEL: an efficient multi-label classifier for recognizing anatomical therapeutic chemical (ATC) classes of drugs. Bioinformatics, 33, 2610. 10.1093/bioinformatics/btz757 31593226

