# Supplementary material for: Drug repurposing against breast cancer by integrating drug-exposure expression profiles and drug–drug links based on graph neural network
Source: Bioinformatics. 2021 Mar 19;37(18):2930–7. doi: 10.1093/bioinformatics/btab191 (PMC8479657; doi:10.1093/bioinformatics/btab191)
Supplement: btab191_Supplementary_Data [file btab191_supplementary_data.zip › Supplementary Material.docx]

Supplementary Material

**Table S3.** Performance based on AUROC and AUPR

| Loss Function  Aggregator |  | AUROC |  |  | AUPR |  |
| --- | --- | --- | --- | --- | --- | --- |
|  | Focal loss  α=0.75  γ=2 | Focal loss  α=0.25  γ=2 | Cross-Entropy | Focal loss  α=0.75  γ=2 | Focal loss  α=0.25  γ=2 | Cross-Entropy |
| GraphSAGE-GCN | **0.81±0.03**  (GraphRepur) | 0.80±0.07 | 0.76±0.08 | **0.59±0.06**  (GraphRepur) | 0.57±0.06 | 0.57±0.04 |
| GraphSAGE -Mean | 0.76±0.05 | 0.77±0.07 | 0.74±0.06 | **0.59±0.09** | 0.58±0.08 | 0.56±0.12 |
| GraphSAGE - LSTM | 0.72±0.18 | 0.68±0.09 | 0.73±0.11 | 0.58±0.08 | 0.55±0.05 | 0.57±0.07 |
| GraphSAGE -MeanPool | 0.79±0.08 | 0.79±0.09 | 0.76±0.09 | 0.57±0.04 | 0.56±0.09 | 0.58±0.05 |
| GraphSAGE -MaxPool | 0.74±0.08 | 0.74±0.08 | 0.73±0.10 | 0.56±0.06 | 0.57±0.05 | 0.55±0.08 |

**Table S4.** Hyperparameters space considered for GCN

| Hyperparameter | Values considered |
| --- | --- |
| Hidden units | (32,64), (64,128), (128,256), (256,512), (32,32), (64,64), (128,128), (256,256), (512,512) |
| Learning rates | 0.01, 0.005, 0.001, 0.0005, 0.0001, 0.00005, 0.00001 |
| Dropout | 0, 0.2, 0.4 |
| Loss function | Focal Loss (α=0.25, γ=2); Focal Loss (α=0.75, γ=2); cross entropy |

**Table S5.** Hyperparameters space considered for deepDR

| Hyperparameter | Values considered |
| --- | --- |
| Batchsize | 8; 16; 32; 64; 128 |
| Learning rates | 0.01, 0.001, 0.0001, 0.00001 |
| Alpha | 2; 4; 8; 16; 32 |
| Beta | 0.1; 0.5; 1; 2; 3 |

**Table S6.** Hyperparameters space considered for BiFusion

| Hyperparameter | Values considered |
| --- | --- |
| Hidden units | (32,64), (64,128), (128,256), (256,512), (32,32), (64,64), (128,128) |
| Learning rates | 0.01, 0.005, 0.001, 0.0005, 0.0001, 0.00005, 0.00001 |
| Batchsize | 8; 16; 32; 64; 128 |

**Table S7.** Hyperparameters space considered for Graph Attention Network

| Hyperparameter | Values considered |
| --- | --- |
| Hidden units | (128 ), (256 ), (512 ), (512, 256 ), (256, 128 ), (128, 64 ), (512, 256, 128 ), (256, 128, 64 ), (128, 64, 32 ), (128, 128, 128 ), (256, 256, 256 ), (512, 512, 512) |
| Learning rates | 0.005, 0.001, 0.0005, 0.0001 |
| Dropout | 0, 0.2, 0.4 |
| The numbers of heads | 8; 4 |
| Loss function | Focal Loss (α=0.25, γ=2); Focal Loss (α=0.75, γ=2); cross entropy |

**Table S8.** Hyperparameters space considered for Deep Neural Network

| Hyperparameter | Values considered |
| --- | --- |
| Hidden units | (128, 64, 1); (512, 256, 1); (1024, 512, 1); (128, 128, 1); (512, 512, 1); (128, 64, 32, 1); (256, 128, 64, 1); (512, 256, 128, 1) |
| Learning rates | 0.001; 0.0001; 0.00001 |
| Dropout | 0.2; 0.4 |
| Batch Size | 32; 64; 128; 256 |
| Loss function | Focal Loss (α=0.25, γ=2); Focal Loss (α=0.75, γ=2); cross entropy |
| Class weight | 1:1; 1:30 |

**Table S9.** Hyperparameters space considered for Support Vector Machines

| Hyperparameter | Values considered |
| --- | --- |
| Regularization parameter C | 0.0001, 0.0005, 0.001, 0.005, 0.01, 1, 10, 100 |
| Kernel coefficient | 0.0001, 0.001, 0.005, 0.01, 1, 10, 100 |
| Kernel type | 'linear', 'sigmoid', 'rbf', 'poly' |

**Table S10.** Hyperparameters space considered for Random Forests

| Hyperparameter | Values considered |
| --- | --- |
| The number of features | 'auto', 'sqrt', 'log2' |
| The maximum depth | 2, 4, 8, 16, 32, 64, 128, 256, 512 |
| Criterion | 'entropy', 'gini' |
| The number of trees | 2, 4, 8, 16, 32, 64, 128, 256, 512 |

**Table S11.** Hyperparameters space considered for Gradient Boosting Machines

| Hyperparameter | Values considered |
| --- | --- |
| The number of boosting stages | 4, 8, 16, 32, 64, 128, 512, 1024 |
| Learning rates | 0.0001, 0.001, 0.01, 0.1, 1 |
| The fraction of samples | 0.3, 0.5, 0.7 |
| Loss function | 'deviance', 'exponential' |

**Table S13.** The performance of GraphRepur on real and random interaction graphs over the five test folds

| Interaction Type | AUROC | AUPR | AUROC (random) | AUPR (random) |
| --- | --- | --- | --- | --- |
| Similarity | 0.7616±0.135 | 0.5712±0.078 | 0.6098±0.134 | 0.5375±0.048 |
| Experimental | 0.6107±0.231 | 0.5465±0.051 | 0.6101±0.047 | 0.5361±0.041 |
| Database | 0.6412±0.178 | 0.5318±0.032 | 0.5961±0.074 | 0.5368±0.038 |
| Text Mining | 0.7861±0.091 | **0.5943±0.039** | 0.6068±0.105 | **0.5371±0.047** |
| Combined Score | **0.8131±0.051** | 0.5857±0.076 | **0.6601±0.078** | 0.5301±0.021 |

**Table S14.** The characteristics of different type interaction graphs

| Interaction Type | Average Node Degree | The Number of Isolated Node |
| --- | --- | --- |
| Similarity | 0.0009 | 592 |
| Experimental | 0.0104 | 334 |
| Database | 0.0001 | 804 |
| Text Mining | 0.0542 | 155 |
| Combined Score | 0.0563 | 155 |

**Table S15.** Performance of GraphSAGE on external validation set

| Method | AUROC | AUPR |
| --- | --- | --- |
| GraphRepur | 0.83±0.10 | 0.63±0.06 |
| GraphSAGE - Mean | 0.82±0.07 | 0.59±0.04 |
| GraphSAGE - LSTM | 0.78±0.12 | 0.60±0.08 |
| GraphSAGE - MeanPool | 0.76±0.09 | 0.57±0.06 |
| GraphSAGE - MaxPool | 0.66±0.12 | 0.55±0.05 |

**Table S18.** Performance of GraphRepur on different cell lines external validation sets

| Cell Line | AUROC | AUPR |
| --- | --- | --- |
| BT-20 | 0.81±0.063 | 0.58±0.056 |
| SK-BR-3 | 0.76±0.145 | 0.58±0.092 |


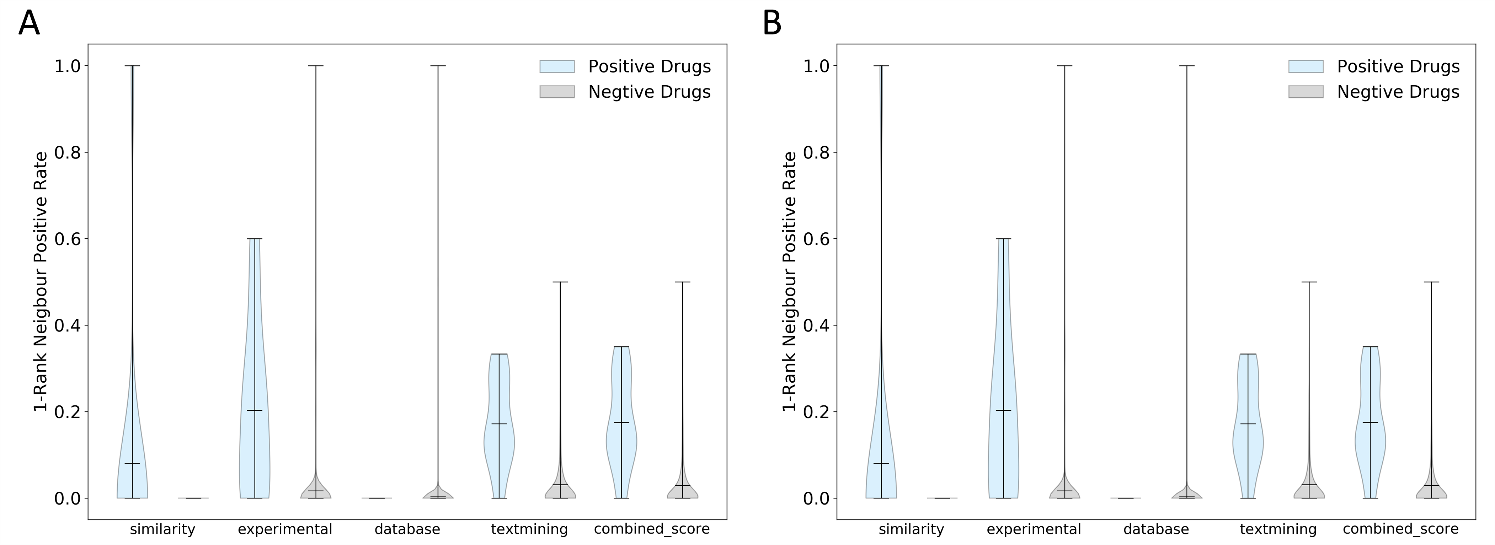


**Figure S1 (A)** Violin plots of the proportion of positive drugs (PPD) in the 1st-order. **(B)** Violin plots of the PPD in 2nd-order neighbor node.
